# Medicinal use of wild fauna by mestizo communities living near San Guillermo Biosphere Reserve (San Juan, Argentina)

**DOI:** 10.1186/1746-4269-11-15

**Published:** 2015-01-21

**Authors:** Jorge Hernandez, Claudia M Campos, Carlos E Borghi

**Affiliations:** Interacciones Biológicas del Desierto (INTERBIODES-CIGEOBIO [Centro de Investigaciones de la Geósfera y la Biósfera, CONICET-UNSJ). Facultad de Ciencias Exactas, Físicas y Naturales, Universidad Nacional de San Juan, Av. Ignacio de la Roza 590 (Oeste), J5402DCS Rivadavia, San Juan, Argentina; Departamento de Biología e Instituto y Museo de Ciencias Naturales, Universidad Nacional de San Juan, Av. España 400 (Norte), 5400 San Juan, Argentina; IADIZA (CONICET), CCT, Mendoza, Argentina

**Keywords:** Ethnozoology, Latin America, Medicinal animals, Mestizaje, Puna, Rural population, Traditional knowledge, Zootherapy

## Abstract

**Background:**

Wild and domestic animals and their by-products are important ingredients in the preparation of curative, protective and preventive medicines. Despite the medicinal use of animals worldwide, this topic has received less attention than the use of medicinal plants. This study assessed the medicinal use of animals by mestizo communities living near San Guillermo MaB Reserve by addressing the following questions: What animal species and body parts are used? What ailments or diseases are treated with remedies from these species? To what extent do mestizo people use animals as a source of medicine? Is the use related to people’s age?

**Methods:**

We conducted semi-structured interviews with 171 inhabitants (15–93 years old) of four villages close to the Reserve: Tudcúm, Angualasto, Malimán and Colangüil. We calculated the informant consensus factor and fidelity level to test homogeneity of knowledge and to know the importance of different medicinal uses for a given species.

**Results:**

The medicinal use of animals was reported by 57% of the surveyed people. Seven species were mentioned: *Rhea pennata, Lama guanicoe, Puma concolor*, *Pseudalopex* sp., *Lama vicugna, Lepus europaeus* and *Conepatus chinga*. Several body parts were used: fat, leg, bezoar-stone, stomach, feather, meat, blood, feces, wool, and liver. The fat of *R. pennata* was the most frequently used animal part, followed by the bezoar stone and the leg of *L. guanicoe.* Animals were used to treat 22 ailments, with respiratory and nervous system disorders being the most frequently treated diseases with a high degree of consensus. Old people used animals as remedies more frequently than young residents, showing some differences among villages.

**Conclusions:**

A low number of animal species was mentioned as used for medicinal purposes, which could be explained by the perception of strong control related the legislation that bans hunting and the erosion of traditional knowledge produced by mestizaje. However, the presence of a traditional medicine is deeply rooted in the community culture. Management strategy for protected areas should focus not only on the conservation and sustainability of biological resources, but also on the ancestral knowledge of local communities, such as the medicinal use of animals.

## Background

The complex past and current relationships between people and natural resources are extremely important to human societies. These interactions can be studied from an ethnobiological perspective, considering, for instance, the use of wildlife for subsistence and commercial purposes [1]. Since ancient times, wild animals and their subproducts have been used for many purposes, such as obtaining food, pets, clothing, adornments, music instruments, etc., and with religious, political or ritual aims [2–4]. Wild and domestic animals and their by-products (e.g., hooves, skins, bones, feathers, tusks) are also important ingredients in the preparation of curative, protective and preventive medicines [5]. This use of animals as remedies is an extremely old practice, probably related to an animal-based diet as well as to the ritual ingestion of the recently deceased in ancient cultures [6]. Thus, animals and products derived from different organs of their bodies have been part of the inventory of medicinal substances used in various cultures, and still persist in traditional medicine. Currently, in modern societies, zootherapy is an important alternative among many other known therapies practiced worldwide [5]; however, this treatment alternative may pose additional pressure over threatened animal populations; thus, there is a need for studies focusing on the use of animals’ body parts as folk medicines to address this conservation issue [7, 8].

In the last 20 years, there has been a notable increase in the number of studies on ethnobiology in Latin America, particularly in Brazil and Mexico, in the area of ethnobotany and involving medicinal plants [1]. In addition, in these countries, as well as in Bolivia, the use of medicinal fauna has been the focus of ethnozoological research e.g. [8–17], although this topic has received less attention than the use of medicinal plants [1, 5]. In Argentina, the use of animals, mainly as food, by native and mestizo populations was assessed in the wet Puna [3], in the Chaco ecoregion [18–23], and in the Monte desert [24].

The present study was conducted in the area surrounding San Guillermo Man and Biosphere Reserve, located in the south of the arid Argentine Puna (San Juan province). This reserve is a biodiversity hot spot in a cold desert, and protects the world’s largest sympatric populations of guanacos (*Lama guanicoe*) and vicuñas (*Lama vicugna*) [25], the pampas cat (*Leopardus colocolo*), the lesser rhea (*Rhea pennata*), the Chilean flamingo (*Phoenicopterus chilensis*), the condor (*Vultur gryphus*), and the horned coot (*Fulica cornuta*), among others [26, 27]. In this region, native American people began to disappear in the 17th century, and by the year 1810 only a mestizo population of Hispanic and Amerindian ancestry was present [28]. Nevertheless, as in other Latin American regions, mestizo people are major users of wildlife [4, 29].

The management strategies for a MaB Reserve, oriented to guarantee conservation and sustainability of the species and ecosystems, require including surrounding local communities [30] and, when possible, their traditional knowledge on the use of wildlife [31]. As for many protected areas including human populations in developing countries, the challenge in San Guillermo Reserve is to achieve biodiversity conservation without negatively affecting local culture. Considering this framework, we assessed the use of animals by mestizo communities living in the area surrounding San Guillermo Reserve by addressing the following questions:

1. What animal species are used as medicinal resources?
2. What body parts are used as a medicine?
3. What ailments or diseases are treated with remedies obtained from these species?
4. To what extent do mestizo people use animals as a source of medicine?
5. Is the medicinal use of animals related to people’s age?

## Methods

### Study area

**Figure 1:**
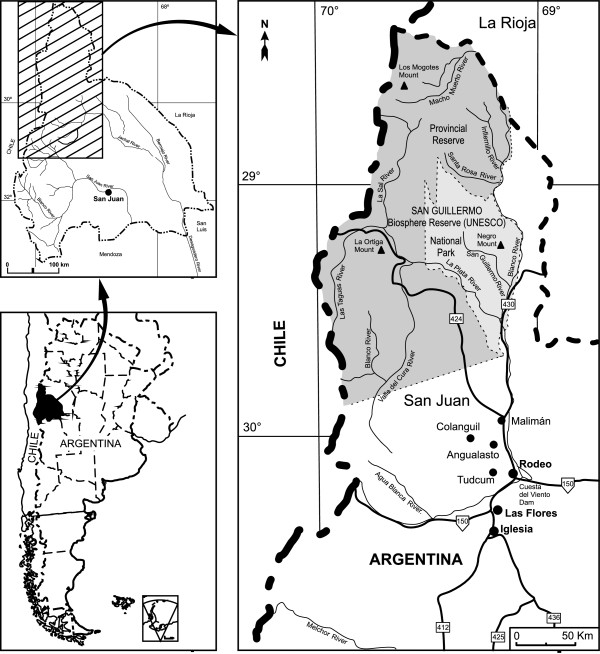
**Communities surrounding San Guillermo MaB Reserve (San Juan, Argentina): Malimán, Colangüil, Angualasto and Tudcúm.**

San Guillermo Man and Biosphere Reserve (29°13′22″S; 69°30′42″W) is located in Iglesias department, northeast of San Juan province, Argentina, to the south of La Rioja province and to the east of Chile (Figure 1).

The Reserve lies mostly in the High Andean and Punean ecoregions, whereas the areas of lowest altitude, where people live, lie in the Monte ecoregion [32]. The flora is mostly Andean and xerophytic [32]. The climate in the valley containing the villages is dry and harsh, with wide diurnal temperature variation and annual range, high solar radiation, exclusively summer rainfall, and winter snowfalls in the Andean area. Annual precipitation is less than 100 mm; annual average temperature is below 18°C dropping to about 0°C at above 3000 m a.s.l., with maximum and minimum absolute temperatures of 25°C and -30°C respectively [33].

### Surrounding communities

There are four villages close to the Reserve: Tudcúm, Angualasto, Malimán and Colangüil, located on the valley of the Blanco River (Figure 1). Tudcúm is the biggest village, with a population of 861 inhabitants, followed by Angualasto, Colangüil, and Malimán, with a 339, 75, and 57 residents, respectively.

Semi-structured questionnaires were used, complemented by free interviews and informal conversations. A total of 171 residents from the villages (Table 1) between 15 years and 93 years old, 59% males and 41% females, were interviewed. The income of 60% of the villagers interviewed are obtained mostly through agriculture, livestock raising, mining, and government employment. Regarding education level, 23 residents (13.45%) had high-school or higher education, 99 (57.90%) had elementary education and 49 (28.65%) received no formal education.

**Table 1:** **Number of people interviewed, divided by gender and the community where they belong**

Community	Gender		Total of surveyed respondents
	Males	Females	
Malimán	9	7	16
Colangüil	11	6	17
Angualasto	25	21	46
Tudcúm	56	36	92
Total	101	70	171

In the anonymous surveys we included key informants of each village (the eldest people, schoolteachers, etc.) based on the information provided by the villagers. According to the locals, their own knowledge of medicinal animals was acquired through parental heritage, or because they had experienced folk medicine healing their kin and/or themselves.

Before being interviewed, local residents were briefed on the research project and its academic objectives. Conversations with inhabitants were based on a common objective: to improve conservation goals of the reserve, include the knowledge regarding traditional use of wildlife in the management of the protected area and develop educational materials of local interest [4], as suggested in the guidelines of the International Society of Ethnobiology Code of Ethics. After that, a verbal informed consent was given by those interviewed, in order to ensure the anonymity of respondents. The interviewed people were asked about the animal species they used as medicine. In order to identify the species accurately and avoid confusion with similar animals, we asked informants to provide the vernacular name by showing pictures that allowed us to identify the scientific name. We inquired about the uses of each animal for treatment of ailments or diseases, the animal body part used, and the method to prepare the remedy. The ailments or diseases treated using animals were grouped into categories according to the responses (Table 2).

**Table 2:** **Animal species used in traditional medicine by mestizo communities living in the area surrounding San Guillermo MaB Reserve (San Juan, Argentina)**

Scientific name/family	English name	Vernacular names	Threat status(IUCN Red List)
*Lama guanicoe* (Müller, 1776)/Camelidae	Guanaco	Guanaco	Least concern
*Lama vicugna* (Molina, 1782)/Camelidae	Vicuña	Vicuña	Least concern
*Rhea pennata* (d’Orbigny, 1834)/Rheidae	Lesser rhea	Avestruz, chure, churi, ñandú	Near Threatened
*Lepus europaeus* (Pallas, 1778)/Leporidae	European hare	Liebre	Least concern
*Puma concolor* (Linnaeus, 1771)/Felidae	Mountain Lion	Puma	Least concern
*Pseudalopex* sp./Canidae	Fox	Zorro	
*Conepatus chinga* (Molina, 1782)/Mephitidae	Molina’s hog-nosed skunk	Chiñe	Least concern

To test homogeneity of knowledge, we used the informant consensus factor [34]:


where Nur refers to the number of use records for a particular use category and Nt refers to the number of taxa used for a particular use category by all informants. Low ICF values (near 0) mean that animals are chosen randomly or that there is no exchange of information about their use among informants; values approaching 1 mean that there is a well-defined selection criterion in the community or information is exchanged among informants [35].

To know the importance of different medicinal uses of a given species reported by informants, we calculated the Fidelity Level (FL) [36]:


based on the ratio between the number of informants who independently suggested the use of a species for the same major purposes (Np) and the total number of informants who mentioned the animal for any use (N). Fidelity level ranges from 1% to 100%, with high FLs (near 100%) being obtained for animals that were used mostly with the same method and low FLs being obtained for species that are used for many different purposes.

## Results

The percentage of population interviewed was as follows: 28% of the population from Malimán, 22.6% from Colangüil, 13.5% from Angualasto and 10.6% from Tudcúm (Table 1). Of the total people surveyed, 57% used animals or their products as remedies.

Seven species (six mammals and one bird) were used for the treatment of different ailments. Table 2 summarizes the scientific and vernacular names of the medicinally used species, and their current conservation status. People used different vernacular names for the animals. Some of them were of Hispanic origin (e.g. liebre), and others were native names (Quechua origin: chure, churi). *Rhea pennata* (lesser rhea) was the species named by the highest number of vernacular names (4), including names of Hispanic and native origin (Table 2).

The species most frequently used by residents were *R. pennata* (58.60% of responses) followed by *L. guanicoe* (guanaco; 44.34%), *Puma concolor* (mountain lion; 5.38%), *Pseudalopex* sp. (fox; 1.07%), *L. vicugna* (vicuña; 0.54%), *L. europaeus* (European hare; 0.54%), and *Conepatus chinga* (Molina’s hog-nosed skunk; 0.54%) (Tables 3 and 4).

**Table 3:** **Medicinal uses of animals and animal parts in traditional therapy in villages close to San Guillermo MaB Reserve (San Juan, Argentina)**

Species	Body part used	Method of preparation and use	Ailment and disease treated	Category	Frequency of responses (N = 186)	Percentages(%)
*Lama guanicoe*	Leg	The leg is left to dry and, when necessary, it is smelled.	Lung diseases	Respiratory system disorders	1	0.54
		The leg is allowed to dry; and then is used to knead the affected area of the body	Cervical muscle spasm (“aire”)	Muscular-skeletal system disorders	1	0.54
		The dry leg is heated over ashes and then it is used to knead the affected area of the body, forming a cross.	Transient facial paralysis (“hora”)	Nervous system disorders	30	16.13
	Feces	The feces are allowed to dry; then hot water is poured over it and the resulting liquid is drunk.	Mountain sickness	Mountain sickness	1	0.54
	Bezoar stone^1^ (Not every guanaco has it)	Found in the stomach or liver of the guanaco, the bezoar stone is bright; immediately after removing the stone from the animal it must be put in the mouth, otherwise it disintegrates. The stone is put in a glass to prepare tea.	Heart diseases	Circulatory system disorders	23	23.36
			Stomach diseases	Digestive system disorders	1	0.54
			Mountain sickness	Mountain sickness	2	1.07
	Wool	Wool is burned to produce smoke or is prepared as incense stick.	Pain in ear in children	Infections	1	0.54
	Meat	Used for cooking food.	High cholesterol level	Circulatory system disorders	1	0.54
	Blood	It is extracted from the animal and drunk.	Mountain sickness	Mountain sickness	1	0.54
*Lama vicugna*	Leg	Same as with guanaco leg. The dry leg is heated over ashes and then it is used to knead the affected area of the body, forming a cross.	Transient facial paralysis (“hora”)	Nervous system disorders	1	0.54
*Rhea pennata*	Fat	It is mixed with lemon juice and sugar, afterwards is heated and then it is drunk. It is melt with tobacco and chamomile and then the preparation is kneaded on the chest, and a hot cloth is put over it. Candies where made of it.	Influenza, cold, chills, and congestion	Infections	28	15.05
			Asthma, bronchitis, and cough	Respiratory system disorders	24	12.90
			Joint and bone pain, rheumatism	Muscular-skeletal system disorders	12	6.45
			Sore throat	Inflammation	5	2.69
			Cervical muscle spasm (“aire”), and spasm in animals	Muscular-skeletal system disorders	1	0.54
	Conti^2^	The lesser rhea stomach is allowed to dry and then it is milled and drunk as a tea or soup. It is also cooked with some water to be used as cream.	Stomach disorder	Digestive system disorders	9	4.84
			Indigestion	Digestive system disorders	9	4.84
			Lung and bronchial problems	Respiratory system disorders	2	1.07
			Cervical muscle spasm (“aire”)	Muscular-skeletal system disorders	1	0.54
			Skin problems	Skin/subcutaneous cellular tissue disorders	1	0.54
	Feather	Smoke is made with a drop of oil or sugar over the embers.	Evil eye	Evil eye	8	4.30
		Smoke is made with a drop of oil or sugar over the embers.	Ear pain in children	Infections	8	4.30
	Meat	To cook food.	High cholesterol level	Circulatory system disorders	1	0.54
*Lepus europaeus*	Fat	The fat is heated, and when cold, the ointment is collected for use.	Lung and bronchial problems.	Respiratory system disorders	1	0.54
*Puma concolor*	Fat	The fat is heated, and when cold, the ointment is collected for use.	Rheumatism and bones’ pain	Muscular-skeletal system disorders	5	2.69
			Chest pain	Respiratory system disorders	5	2.69
*Pseudalopex* sp.	Fat	The fat is heated, and when cold, the ointment is collected for use.	Asthma	Respiratory system disorders	2	1.07
*Conepatus chinga*	Liver	It is allowed to dry and then it is drunk as a tea or soup.	Lung problems	Respiratory system disorders	1	0.54

^1^ Bezoar stone: The first approximation is that the bezoar stone is a silicate’s vesicle calculus of *Lama guanicoe* (Osvaldo Olivera; personal comm.).

^2^ Conti: *Rhea pennata*’s stomach.

**Table 4:** **Ailment and disease categories with their informant consensus factor (ICF), the species preferred for the treatment of this disorder, fidelity level (FL), and percentage of responses**

Ailment and disease categories	ICF	Preferred species	FL	Percentage of responses
Respiratory system disorders	0.86	*Rhea pennata*	31.32	13.97
Digestive system disorders	0.94	*Rhea pennata*	19.78	9.68
Muscular-skeletal system disorders	0.89	*Rhea pennata*	14.74	7.53
Nervous system disorders	0.97	*Lama guanicoe*	93.75	16.13
Skin/subcutaneous cellular tissue disorders	0	*Rhea pennata*	0.93	0.54
Inflammation	1	*Rhea pennata*	4.81	2.69
Infections	1	*Rhea pennata*	49.31	19.35
Circulatory system disorders	0.96	*Lama guanicoe*	38.71	23.90
Mountain sickness	1	*Lama guanicoe*	6.45	2.15
Evil eye	1	*Rhea pennata*	7.92	4.30

**Figure 2:**
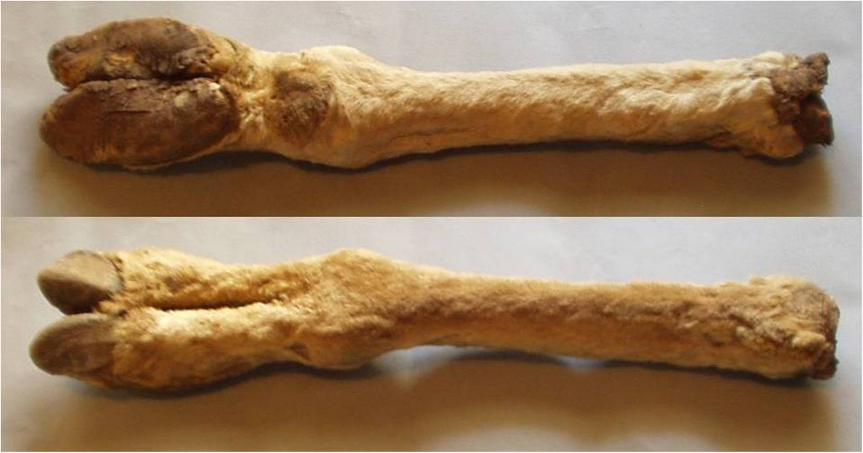
**Guanaco leg used as medicinal resource.**

Table 3 summarizes the body part used as medicinal product, the method of preparation and administration, the diseases or ailments treated, and the frequency and percentages of responses. Several parts of the animals were used for medical reasons: fat, leg, bezoar stone, stomach, feather, meat, blood, feces, wool, and liver. The fat of *R. pennata* was the most frequently used animal part (37.63% of responses), followed by the bezoar stone and the leg of *L. guanicoe* (24.93% and 17.21% of responses, respectively; Table 3 and Figure 2).

Animal parts or products were reported to be used to treat 22 ailments or diseases: joint and bone pain, asthma, bronchial problems, cervical muscle spasm, cold, cough, chills, chest pain, congestion, earache, heart diseases, high cholesterol level, indigestion, influenza, lung diseases, mountain sickness, facial paralysis, rheumatism, skin problems, sore throat, and stomach disorders. Some species were recorded as having magic use, such as the cure of evil eye (Table 2). Table 4 shows the 10 categories that include the ailments and diseases named by respondents.

The categories with most frequent use records were those of animals used for treatment of respiratory system disorders (36 use records, 6 species), nervous system disorders (31 use records, 2 species), infections (28 use records, 1 species), circulatory system disorders (25 use records, 2 species), and muscular-skeletal system disorders (20 use records, 3 species). All these categories had a high degree of consensus, with ICF values greater than 0.80 (Table 4). The category of animals used to treat skin/subcutaneous cellular tissue disorders had the lowest degree of consensus (ICF = 0); only one informant mentioned ailments in this category and used only one species to treat them.

Some animal species were widely used for specific therapeutic purposes, showing a high percentage of responses accompanied by a high fidelity level. For instance, *L. guanicoe* was used for the treatment of nervous and circulatory systems disorders, and *R. pennata* was used for infections and respiratory system disorders (Table 4).

Some animal parts were used for a variety of ailments and diseases, whereas other parts were used to treat one specific disorder. The fat and the stomach of *R. pennata* were claimed to be used for five and four ailments, respectively. The fats of other species, such as *P. concolor, L. europaeus,* and *Pseudalopex* sp., were used to treat specific ailments related to respiratory disorders, and muscular-skeletal system diseases (Table 3).

**Figure 3:**
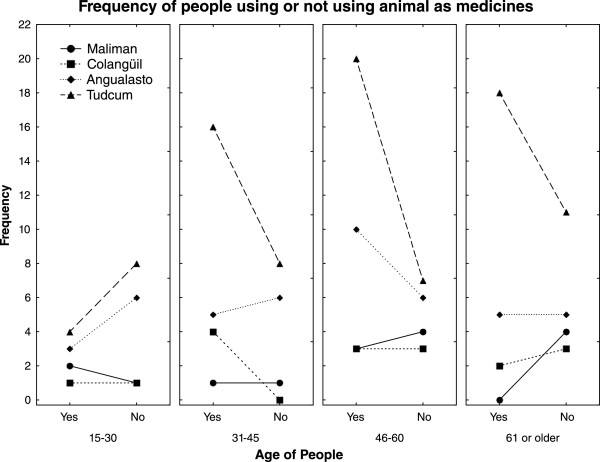
**Frequencies of people reporting medicinal use of animals in the four villages surrounding San Guillermo MaB Reserve (San Juan, Argentina).**

In general, older people used animals as remedies more frequently than younger residents (Figure 3); nevertheless, there were differences among villages. For instance, in Tudcum, residents from the age of 31 onwards indicated a high use of animals as medicines, whereas in Malimán, the village closest to the Reserve, the reported use was low at all ages (Figure 3).

## Discussion

The results clearly indicate the presence of a traditional medicine deeply rooted in the community culture, coexisting with the conventional medicine offered through a hospital located in a close city (Rodeo; 34 km from the farthest village), and through health care centres, run by physicians and sanitary agents, located in Tudcum and Angualasto. In this diverse sanitary context, the number of used species recorded in the present study (seven species) was slightly higher than the number reported by rural populations in drylands of Argentina, such as the Chaco ecoregion (four species) [20], and lower than the numbers used by indigenous population in the Chaco (15 and 72 species, respectively) [21, 22], in the Puna (17 species) [3], and in the semi-arid region of the northeastern Brazil (25 species); [11]. Nevertheless, the seven species reported as used in our study represented 30% of the mammal diversity of San Guillermo MaB Reserve [4, 26]. Among the causes of the low number of animals used here reported could be the restrictive legislation related to hunting and the mestizaje of population.

The prohibition of hunting by the current provincial legislation (provincial Law No. 6,911) could be preventing people from sharing knowledge and information with researchers, as reported in Brazilian studies [1, 37], even when the surveys were anonymous. The smaller villages are closer to the Reserve than the larger ones. In addition, their residents reported the lower medicinal use of animals, probably because they perceive strong government control. In the last decade, at the country level, legal regulations to guarantee wildlife conservation were enforced; today, the most feasible options for sustainable economic wildlife utilization seem to be the live shearing of *L. guanicoe* and the use of *Rhea* spp. products obtained from captive-bred animals. Since profitability of live shearing of *L. guanicoe* depends on the number of animals captured and sheared, this activity should be restricted to areas in which wild populations are abundant, such as in the south of Mendoza, east of Neuquén and southeast of Río Negro provinces. Hunting of *Rhea* spp. was prohibited in 1986, and the commerce of its products was authorized in some provinces in 2000, only when ranching techniques are employed and registered farms are involved. However, in San Juan province there are no authorized farms processing any wild animal for human consumption.

European colonization modified the relationship between dwellers and native species of the arid zones of Argentina through the commercial use of natural resources, the implementation of agriculture and animal husbandry, and the introduction of exotic crop and weed species [24]. The mestizaje between native and Hispanic populations can still be detected in the use of native and/or Hispanic names for native and domestic animals, and probably also influenced the erosion of traditional knowledge and practices involving medicinal use of animals.

The erosion of traditional knowledge has also possibly been aggravated by the expansion of modern education, which has contributed to undermining traditional values among the young people [38–40]. Traditional knowledge, mainly transmitted orally, may be vulnerable to extinction because aged residents are dying, with their knowledge left unrecorded and new generations have learned little from them [38, 41, 42].

In our study, the use of animal fat represented 44.62% of use records, involving several species, such as *R. pennata, L. europaeus, P. concolor, Pseudalopex* sp., and *C. chinga.* These findings are consistent with other studies indicating that the use of animal fat in folk medicine is a common phenomenon [9, 15, 43–46].

Accessibility and availability of local faunal resources influence the choices of the zootherapeutics utilized [45, 11]. Particularly, the fat of *R. pennata* (37.7%), the bezoar stone (25%) and the leg (17.21%) of *L. guanicoe* were the most frequently used animal parts in the area, possibly because these species live near the villages and are traditionally the most familiar to the people, and the most heavily hunted (authors’ personal observations). These species are also utilized as food (4), reinforcing the importance of wild animals as a resource of medicinal and nutritional products, and emphasizing the need for a sustainable use of biodiversity. This result is consistent with recent field investigations in other parts of the world [9, 47].

According to other studies, the category with the largest number of citations was treatment of respiratory system disorders. In Brazil, zootherapeutics are also most frequently used to treat the common illnesses in the population, such as problems affecting the respiratory apparatus (including throat inflammations, coughing, colds, and asthma) [45, 46, 48, 49].

Furthermore, after using the guanaco leg as a remedy in a family, the housewife dries it and shares it with neighbors for the treatment of illnesses. The same happens with the fat of *R. pennata* and *P. concolor*. Solidarity between neighbors and cultural transmission of the use of fauna with medical purposes would help keep this tradition alive from generation to generation.

The results obtained in the present research agree with data provided by other studies, and confirm that the medicinal use of animals shows an important connection between people and nature. The research about popular knowledge applied to zootherapeutic practices offers the opportunity to conciliate efforts directed to conserve cultural and biological diversity.

## Conclusions

In a context where traditional and conventional medicine coexist, the use of animals to treat ailments by mestizo communities in the mountain desert of San Juan is an evidence of cultural inheritance left by the native people, which has survived despite legal regulations and miscegenation. Biodiversity conservation involves nature and people; therefore, it should not imply “harassing” the manifestations of traditional knowledge, in this case belonging to people from the villages surrounding the San Guillermo Biosphere Reserve.

Some species were familiar to people and widely used as medicinal resources. Seven species were mentioned as used to treat approximately 22 ailments or diseases, mainly related to respiratory and nervous system disorders. Species such as *L. guanicoe* and *R. pennata* were widely used for therapeutic purposes. Some animal parts were used for a variety of ailments and diseases, such as the fat and the stomach of *R. pennata.*

Elderly people claimed to use animals as remedies more intensively than younger people did, but the declared intensity of use and the differences between ages differed among villages. The greatest use of animals as medicines corresponded to the biggest village, which was the most distant from the Reserve, whereas people inhabiting the smallest and closest village reported the lowest medicinal use of animals. These results could reflect the perception of control, mainly in the area close to the protected area, because animal hunting is not allowed by the provincial legislation.

In summary, our findings highlight the importance of including the ancestral knowledge of local communities, such as the medicinal use of animals, in the implementation of management strategies for protected areas, especially the UNESCO Man and Biosphere ones, which should be oriented to guarantee the environmental sustainability and conservation of biological and cultural goods.
